# Targeting PIM kinase enhances the activity of sunitinib in renal cell carcinoma

**DOI:** 10.1038/bjc.2011.426

**Published:** 2011-10-20

**Authors:** D Mahalingam, C M Espitia, E C Medina, J A Esquivel, K R Kelly, D Bearss, G Choy, P Taverna, J S Carew, F J Giles, S T Nawrocki

**Affiliations:** 1 Department of Medicine, Institute for Drug Development, Cancer Therapy and Research Center, The University of Texas Health Science Center, 7979 Wurzbach Road, San Antonio, TX 78245, USA; 2 SuperGen Inc., Dublin, CA 94568, USA

**Keywords:** PIM, SGI-1776, sunitinib, renal cell carcinoma, c-Myc

## Abstract

**Background:**

Upregulation of PIM kinase expression has been reported in many malignancies, suggesting that inhibition of PIM kinase activity may be an attractive therapeutic strategy. We hypothesised that inhibition of PIM kinase activity with SGI-1776, a novel small molecule inhibitor of PIM kinase activity, would reduce the viability of renal cell carcinoma (RCC) cells and enhance the activity of sunitinib.

**Methods:**

Immunoblotting, qRT–PCR, and gene expression arrays were carried out to identify genes modulated by SGI-1776 treatment. The anticancer activity of SGI-1776 and sunitinib was determined by viability and apoptosis assays and in tumour xenografts *in vivo*.

**Results:**

Treatment with SGI-1776 led to a decrease in phosphorylated and total c-Myc levels, which resulted in the modulation of c-Myc target genes. SGI-1776 in combination with sunitinib induced a further reduction in c-Myc levels, which was associated with enhanced anticancer activity. siRNA-mediated knockdown of c-Myc demonstrated that its expression has a key role in regulating the sensitivity to the combination of SGI-1776 and sunitinib. Importantly, the combination significantly reduced tumour burden in two RCC xenograft models compared with single-agent therapy and was very well tolerated.

**Conclusion:**

These data indicate that targeting PIM kinase signalling is a promising treatment strategy for RCC.

Renal cell carcinoma (RCC) accounts for 85% of kidney cancers with clear cell RCC (ccRCC) being the most common subtype representing ∼75% of all cases (Kaelin, 2008). Loss of expression of the von Hippel-Lindau (VHL) tumour suppressor causes stabilisation of hypoxia-inducible factors (HIFs) and occurs in 70% of sporadic ccRCC patients (Kaelin, 2007). Hypoxia-inducible factors alter the cellular environment by activating target genes involved in angiogenesis and metabolism (Turner *et al*, 2002). The anti-angiogenic multi-tyrosine kinase inhibitors sunitinib and sorafenib demonstrated efficacy for the treatment of RCC likely due to the highly vascularised nature of these tumours. Despite the success of these agents, drug resistance is a major obstacle that highlights the need for new treatment strategies to improve clinical outcomes (Sosman *et al*, 2007).

The PIM kinases are a family of serine/threonine kinases (*PIM-1*, *PIM-2*, *PIM-3*) that have been associated with tumourigenesis and drug resistance (Adam *et al*, 2006; Cibull *et al*, 2006; Beier *et al*, 2007; Chen *et al*, 2008, 2009a). All three kinases have an ATP-binding pocket, an active site, a kinase domain, and lack regulatory domains making them constitutively active. The expression of PIM kinases is mediated by the Janus-activated kinase/signal transducers and activators of transcription (STAT) signalling pathway (Amaravadi and Thompson, 2005). PIM kinases regulate cell-cycle progression by directly phosphorylating p21, p27, Cdc25A, and Cdc25C and suppress apoptosis via phosphorylation of the pro-apoptotic protein Bad (Mochizuki *et al*, 1999; Li *et al*, 2006; Popivanova *et al*, 2007; Zhang *et al*, 2007b; Morishita *et al*, 2008; Hu *et al*, 2009). Overexpression of PIM-1 has been reported in haematological malignancies and in many solid tumours, which suggests that blocking PIM-1 kinase activity may be a promising approach for cancer therapy (Eichmann *et al*, 2000; Cibull *et al*, 2006; Beier *et al*, 2007; Chen *et al*, 2008; Hogan *et al*, 2008).

While PIM-1 is not a potent oncogene, it interacts synergistically with c-Myc to promote tumourigenesis (Moroy *et al*, 1991). Phosphorylation of c-Myc by PIM-1 leads to c-Myc stabilisation and enhanced transcriptional activity (Zippo *et al*, 2007; Zhang *et al*, 2008). Given the central role of Myc in many cancers, including RCC, inhibition of PIM-1 activity may promote Myc degradation and subsequently decrease tumour cell proliferation (Pelengaris *et al*, 2002; Nilsson and Cleveland, 2003; Tang *et al*, 2009). This approach may be especially effective in a subset of VHL-deficient RCCs that express HIF-2*α*, but not HIF-1*α*, which promotes elevated c-Myc activity (Gordan *et al*, 2007a, 2008).

In this study, we evaluated the efficacy of a novel small molecule inhibitor of PIM kinase activity, SGI-1776, in RCC alone and in combination with sunitinib. SGI-1776 demonstrated activity in a panel of RCC cell lines, which was associated with decreased phosphorylation of the PIM kinase substrates, Bad and c-Myc. Furthermore, SGI-1776 reduced total protein levels of c-Myc and microarray analyses displayed gene expression patterns consistent with blunted c-Myc activity. Importantly, targeting PIM-1 with either siRNA or SGI-1776 significantly enhanced the activity of sunitinib in RCC models. Our results establish that SGI-1776 decreases c-Myc levels, reduces RCC viability, and enhances the activity of sunitinib. Collectively, these findings indicate that inhibition of PIM kinase activity in combination with sunitinib warrants further investigation for the treatment of RCC and other malignancies.

## Materials and methods

### Cells and cell culture

786-O, A498, Caki-1, Caki-2, and ACHN renal cancer cell lines were obtained from the American Type Culture Collection (Manassas, VA, USA). Cancer cell lines were cultured in RPMI supplemented with 10% fetal bovine serum and maintained in a humidified incubator at 37 °C with 5% CO_2_. Human normal renal proximal tubule epithelial cells (RPTECs) were purchased from Clonetics (Walkersville, MD, USA) and cultured in the recommended media (REGM BulletKit, Clonetics).

### Antibodies and reagents

Antibodies were obtained from the following commercial sources: anti-PIM-1 (Santa Cruz Biotechnology, Santa Cruz, CA, USA); anti-phospho-Bad (Ser112) and Bad (Cell Signaling, Danvers, MA, USA); anti-actin and tubulin (Sigma-Aldrich, St Louis, MO, USA); anti-c-Myc, phospho-c-Myc (Ser62), and Bad (immunohistochemistry) (Novus Biologicals, Littleton, CO, USA); antiproliferating cell nuclear antigen (PCNA) (Dako, Glostrup, Denmark); goat anti-rabbit horseradish peroxidase (HRP)-conjugated secondary antibodies (Jackson Laboratories, West Grove, PA, USA); Rat anti-mouse IgG2a-HRP (Serotec, Raleigh, NC, USA); and sheep anti-mouse-HRP and donkey anti-rabbit-HRP (Amersham, Pittsburgh, PA, USA). SGI-1776 was kindly provided by SuperGen Inc. (Dublin, CA, USA). Sunitinib was purchased from the Cancer Therapy and Research Center pharmacy.

### Quantification of drug-induced cytotoxicity

Cell viability was assessed by 3-(4,5-dimethylthiazol-2-yl)-2,5-diphenyltetrazolium bromide (MTT) assay and the pro-apoptotic effects of SGI-1776 and sunitinib were quantified by propidium iodide (PI) staining and fluorescence-activated cell sorting (FACS) analysis of sub-G_0_/G_1_ DNA content as previously described (Carew *et al*, 2009).

### Clonogenic survival assays

Clonogenic survival assays were conducted as previously described (Mahalingam *et al*, 2010). Briefly, cells were treated with 5 *μ*M SGI-1776, 5 *μ*M sunitinib, or the combination for 24 h. After drug exposure, cells were washed twice in PBS followed by the addition of fresh media and incubated for 10 days in a humidified incubator at 37 °C with 5% CO_2_. Colonies were then washed in PBS, fixed with methanol, and stained with crystal violet. Colonies were scored using an Alpha Innotech (San Leandro, CA, USA) gel documentation system.

### Immunoblotting

Renal cell carcinoma cells were treated with the indicated concentrations of drugs. Immunoblotting was performed as previously described (Nawrocki *et al*, 2008). Quantification of bands was performed using ImageJ software (Bethesda, MD, USA).

### Transfection of siRNAs

PIM-1, c-Myc, and non-target SMARTpool siRNA were obtained from Dharmacon (Lafayette, CO, USA). Cells were transfected with 100 nM of each siRNA using Oligofectamine (Invitrogen, Carlsbad, CA, USA) according to the manufacturer's protocol. Transfected cells were incubated at 37 °C for 24 h and then treated with SGI-1776, sunitinib, or the combination for 48 h. Efficiency of RNAi was measured at 48 h by immunoblotting.

### Expression microarrays

786-O and Caki-1 cells were treated with 5 *μ*M SGI-1776 for 24 h. Following drug treatment, total RNAs were isolated using the RNeasy Plus Mini Kit (Qiagen, Germantown, MD, USA) and treated with TURBO DNA-*free* Kit (Applied Biosystems, Foster City, CA, USA). In all, 300 ng of total RNA per sample was amplified and hybridised to GeneChip Human Gene 1.0 ST arrays (Affymetrix, Inc., Santa Clara, CA, USA) according to the manufacturer's instructions. Affymetrix CEL files were imported into Partek Genomics Suite 6.4 (Partek Inc., St Louis, MO, USA) using the default Partek normalisation parameters and the robust multi-array average (RMA) analysis adjusted for probe sequence and GC content (GC-RMA). Data normalisation was performed across all arrays using quantile normalisation.

### Quantitative real-time polymerase chain reaction

786-O and Caki-1 cells were treated with 5 *μ*M SGI-1776 for 24 h. cDNA from control and SGI-1776-treated cells were used for relative quantification by real-time polymerase chain reaction (RT–PCR) analyses. First-strand cDNA synthesis was performed from 1 *μ*g RNA in a 20-*μ*l reaction mixture using the high-capacity cDNA Reverse Transcription Kit (Applied Biosystems). *CDC25A*, *ODC1*, *SKP2*, *CDKN1A*, *DDIT3*, *PIM-1*, and *GAPDH* transcripts were amplified using commercially available TaqMan Gene expression assays (Applied Biosystems). Relative gene expression was calculated with the 2^−ΔΔCt^ method using *GAPDH* as a housekeeping gene (Pfaffl, 2001).

### Xenograft studies

All experiments were conducted in accordance with the guidelines for the welfare and use of animals in cancer research (Workman *et al*, 2010). Animal protocols were approved by the Institutional Animal Care and Use Committee of the University of Texas Health Science Center at San Antonio. 786-O and Caki-1 renal cancer cells (1 × 10^7^) were suspended in a mixture of HBSS and Matrigel (BD BioSciences, San Jose, CA, USA) and subcutaneously implanted into female nude mice (BALB/c background) from Harlan (Indianapolis, IN, USA). Tumour-bearing animals from each cell line xenograft were randomised into treatment groups. Mice were treated with vehicle, sunitinib (40 mg kg^–1^ PO), SGI-1776 (200 mg kg^–1^ free base PO), or both agents on a QDx5 (every day for 5 days) schedule for 3 weeks. Mice were monitored daily and tumour volumes were measured twice weekly.

### Immunohistochemistry

Paraffin-embedded tumour sections were deparaffinised in xylene, treated with a graded series of alcohol, and rehydrated in PBS (pH 7.5). Heat-induced epitope retrieval was performed by microwaving slides in a citrate buffer. Primary antibodies were added and slides were incubated at 4 °C overnight. After washing with PBS, slides were incubated in appropriate secondary antibodies for 1 h at ambient temperature. Positive reactions were visualised by immersing the slides with stable 3,3-diaminobenzidi (Research Genetics, Huntsville, AL, USA) and counterstained with Gill's haematoxylin (Sigma). Images were captured using an Olympus fluorescent microscope (Center Valley, PA, USA) with a DP71 camera and a × 20 objective. Image-Pro Plus software Version 6.2.1 (MediaCybernetics, Bethesda, MD, USA) was used for image acquisition and quantification by densitometric analysis of five random fields containing viable tumour cells.

### Terminal deoxyribonucleotide-transferase-mediated dUTP nick-end labelling assay

DNA fragmentation was analysed using a FITC-labelled TUNEL assay kit (Promega, Madison, WI, USA). The assay was performed according to the manufacturer's instructions and PI was used to counterstain the nucleus. Percentages of TUNEL-positive cells were determined by manual counting of five random fields per section.

### Statistical analyses

Statistical significance was determined using the Tukey–Kramer comparison test or the Student's *t*-test. Differences were considered significant in all experiments at *P*<0.05.

## Results

### PIM-1 expression is increased in RCC cells

**Figure 1:**
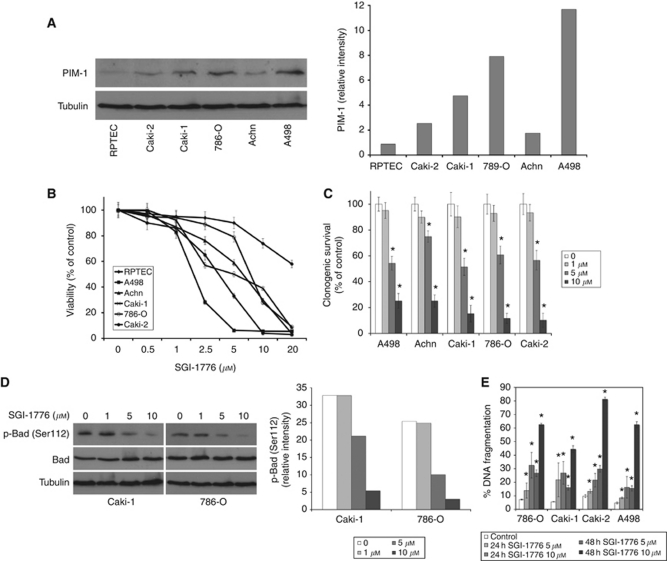
PIM-1 is overexpressed in RCC cell lines and inhibition of PIM kinase activity with SGI-1776 promotes apoptosis and reduced viability. (**A**) PIM-1 kinase expression in RCC cell lines. Immunoblotting demonstrates elevated PIM-1 levels compared with normal RPTECs. Band intensity was quantified using ImageJ software. (**B**) SGI-1776 reduces RCC cell viability. Five RCC cell lines and RPTEC cells were incubated with the indicated concentrations of SGI-1776 for 72 h and cell viability was measured by MTT assay. Mean±s.d., *n*=3. (**C**) SGI-1776 reduces RCC clonogenic survival. Cells were treated with indicated concentrations of SGI-1776 for 24 h, washed and incubated in fresh media for 10 days. Colonies were fixed, stained with crystal violet and quantified. Mean±s.d., *n*=3. ^*^Indicates a significant difference compared with control. *P*<0.05. (**D**) SGI-1776 decreases Bad phosphorylation at Ser112. Cells were treated with SGI-1776 for 48 h and phospho- and total Bad levels were determined by immunoblotting. Phospho-Bad band intensity was quantified using ImageJ software. (**E**) SGI-1776 induces apoptosis in RCC cell lines. Cells were treated with SGI-1776 for 48 h and apoptosis was measured by PI-FACS analysis. Mean±s.d., *n*=3. ^*^Indicates a significant difference compared with control. *P*<0.05.

Upregulation of PIM kinase expression has been observed in many malignancies, but has not been investigated in RCC. Considering the proposed role of PIM-1 in tumourigenesis and drug resistance, we evaluated its expression in a panel of RCC cell lines and in normal RPTECs. PIM-1 displayed varying degrees of expression in RCC cell lines, but was consistently increased in all lines compared with normal RPTECs (Figure 1A).

### The PIM kinase inhibitor SGI-1776 reduces RCC viability

We next evaluated the ability of the small molecule PIM kinase inhibitor SGI-1776 to reduce RCC viability. The MTT assays demonstrated that SGI-1776 exhibited activity against all of the RCC lines tested with less toxicity against normal RPTEC cells (Figure 1B). Furthermore, SGI-1776 also effectively reduced RCC cell clonogenic survival (Figure 1C). PIM kinases have been previously reported to phosphorylate the pro-apoptotic protein Bad at Ser112, which promotes its sequestration in the cytosol by 14-3-3 and thus, blocks its mitochondrial interaction with Bcl-2 or Bcl-X_L_ (Fox *et al*, 2003; Yan *et al*, 2003; Aho *et al*, 2004; Li *et al*, 2006). Therefore, we hypothesised that inhibition of PIM kinase activity may stimulate apoptosis by freeing Bad to interact with anti-apoptotic proteins. As expected, abrogation of PIM kinase activity reduced Bad phosphorylation at Ser112 (Figure 1D) and induced apoptosis in a panel of RCC cell lines (Figure 1E).

### SGI-1776 inhibits c-Myc phosphorylation and modulates the expression of c-Myc target genes

**Figure 2:**
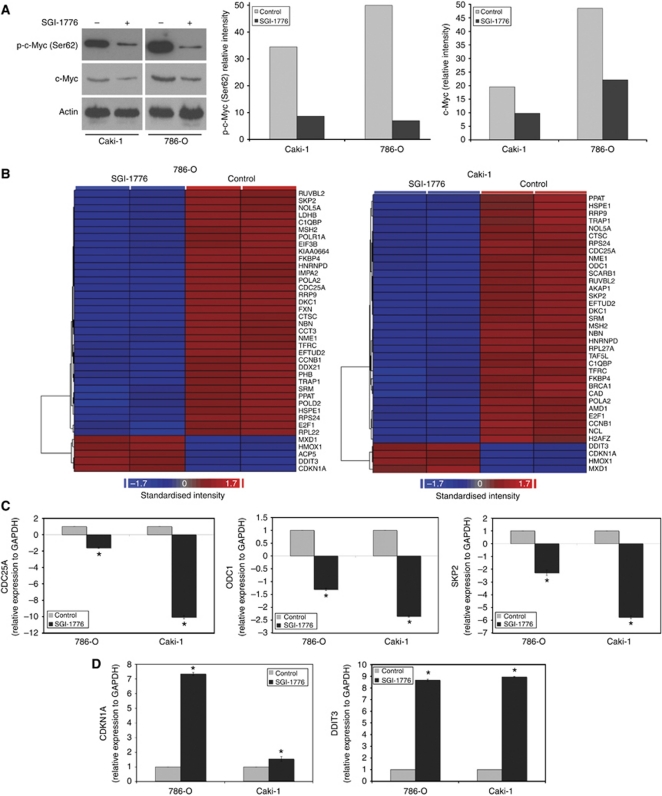
SGI-1776 decreases phospho- and total c-Myc levels, resulting in altered expression of c-Myc target genes. (**A**) SGI-1776 reduces functional c-Myc levels. RCC cells were treated with 5 *μ*M SGI-1776 for 48 h. Phospho- and total c-Myc levels were measured by immunoblotting. Phospho-c-Myc and c-Myc band intensity was quantified using ImageJ software. (**B**) Affymetrix expression arrays identify differential expression of c-Myc target genes following 24 h treatment with 5 *μ*M SGI-1776. Blue corresponds to reduced gene expression and red corresponds to increased expression. RNA isolation and expression arrays were performed as described in Materials and methods. (**C** and **D**) qRT–PCR analysis of selected genes that are upregulated (*CDC25A*, *ODC1*, and *SKP2*) or downregulated (*CDKN1A* and *DDIT3*) by c-Myc. Cells were treated with 5 *μ*M SGI-1776 for 24 h and then harvested for analysis. Levels of mRNAs were standardised to the expression of *GAPDH*. Mean±s.d., *n*=3. ^*^Indicates a significant difference from the controls. *P*<0.05.

In addition to Bad, another target of PIM kinase phosphorylation is the oncogene c-Myc. Overexpression of PIM kinases are frequently associated with elevated Myc levels. Since c-Myc has an important role in the biology of RCC, we determined the effect of SGI-1776 on its phosphorylation status in the 786-O and Caki-1 cell lines. Treatment with SGI-1776 induced a strong reduction in c-Myc phosphorylation at Ser62 (Figure 2A). In accordance with the reports that this phosphorylation site promotes protein stability, SGI-1776 exposure also resulted in a decrease in total c-Myc protein expression (Figure 2A). Expression profiling (Figure 2B) and qRT–PCR (Figures 2C and D) analyses of RNA isolated from SGI-1776-treated 786-O and Caki-1 cells showed significant changes in the levels of c-Myc target genes that were consistent with reduced c-Myc activity. c-Myc behaves as both a transcriptional activator and repressor, inducing transcription of genes (e.g., *CDC25A*, *ODC*, and *SKP2*) by binding to CACGTG regions in a complex with Max, and blocking the transcription of other genes (e.g., *CDKN1A*) while complexed with Max and Miz1 or Sp1 (Nilsson and Cleveland, 2003; Gordan *et al*, 2007b). Collectively, our results suggest that inhibition of PIM kinase activity with SGI-1776 decreases functional c-Myc protein levels and corresponding transcriptional activity.

### Inhibition of PIM-1 activity enhances the activity of sunitinib in RCC

**Figure 3:**
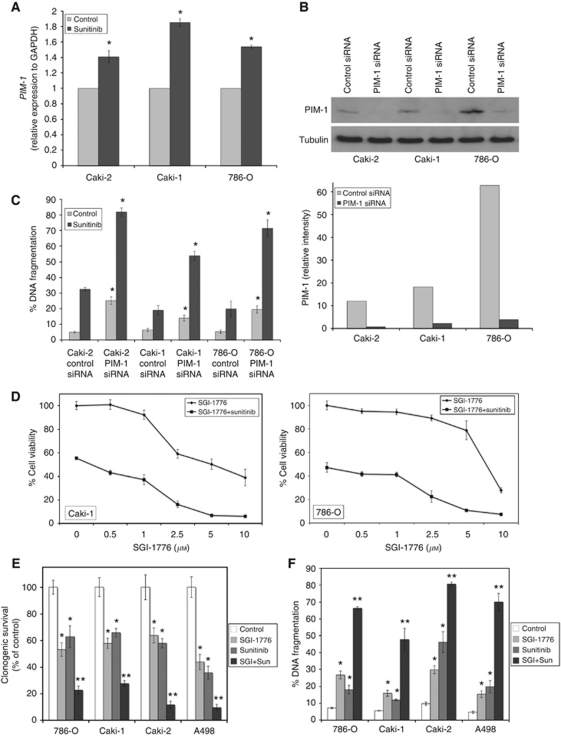
Inhibition of PIM-1 kinase activity augments sunitinib-induced cell death. (**A**) Sunitinib induces *PIM-1* expression. Cells were treated for 24 h with 5 *μ*M sunitinib and *PIM-1* levels were measured by qRT–PCR. *PIM-1* expression was standardised to *GAPDH*. Mean±s.d., *n*=3. ^*^Indicates a significant difference from the controls. *P*<0.05. (**B**) Silencing PIM-1 expression using siRNA. Cells were transfected with control or PIM-1 siRNA and PIM-1 expression levels were determined by immunoblotting. Band intensity was quantified using ImageJ software. (**C**) Knockdown of PIM-1 sensitises RCC cells to sunitinib-induced apoptosis. Cells were transfected with control or PIM-1 siRNA. After transfection, cells were treated with 5 *μ*M sunitinib for 48 h. Apoptosis was measured by PI-FACS analysis. Mean±s.d., *n*=3. ^*^Represents a significant difference compared with control siRNA-transfected cells. (**D**) SGI-1776 in combination with sunitinib reduces RCC cell viability. Cells were incubated with increasing concentrations of SGI-1776 with 5 *μ*M sunitinib for 72 h and cell viability was measured by MTT assay. Mean±s.d., *n*=3. Note: Single-agent sunitinib treatment is represented by the 0-*μ*M SGI-1776 concentration of the SGI-1776+sunitinib group. (**E**) SGI-1776 augments the effects of sunitinib on clonogenic survival. RCC cells were treated with 5 *μ*M SGI-1776, 5 *μ*M sunitinib, or both agents for 24 h. Cells were washed and incubated in fresh media for 10 days. Colonies were then fixed, stained with crystal violet, and quantified. Mean±s.d., *n*=3. (**F**) SGI-1776 enhances sunitinib-induced apoptosis. Apoptosis was determined by PI-FACS analysis. Mean±s.d., *n*=3. ^*^Indicates a significant difference compared with controls and ^**^indicates a significant difference compared with either single-agent treatment. *P*<0.05.

The multi-tyrosine kinase inhibitor sunitinib is given as first-line therapy for the treatment of metastatic RCC. Although sunitinib has demonstrated clinical benefit in RCC, drug resistance continues to be a major obstacle (Heng and Bukowski, 2008). Sunitinib treatment stimulated a significant increase in *PIM-1* expression levels, suggesting that PIM-1 activity may promote resistance to sunitinib-induced apoptosis (Figure 3A). In order to further investigate this possibility, we utilised siRNA to knockdown PIM-1 levels (Figure 3B). This demonstrated that targeted PIM-1 knockdown significantly sensitised RCC cells to sunitinib (Figure 3C). Consistent with this result, addition of SGI-1776 to sunitinib markedly decreased cell viability in RCC cell lines (Figure 3D). We next evaluated the effects of SGI-1776 and sunitinib on clonogenic survival and apoptosis. Importantly, the combination significantly reduced clonogenic survival (Figure 3E) and induced apoptosis (Figure 3F) in a panel of RCC cell lines.

### Treatment with SGI-1776 and sunitinib reduces c-Myc levels

**Figure 4:**
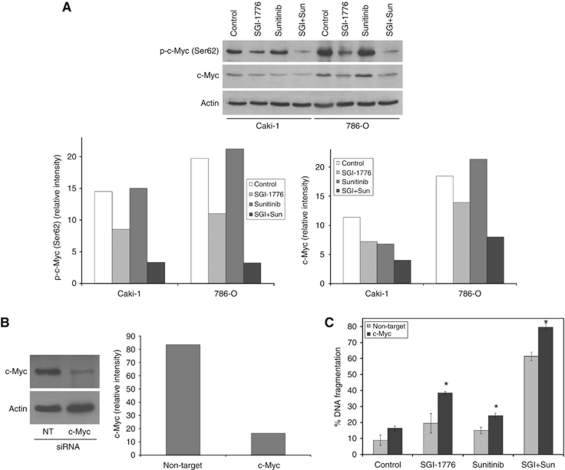
Knockdown of c-Myc levels augments the activity of SGI-1776 and sunitinib. (**A**) The combination of SGI-1776 and sunitinib stimulates a further reduction in phosphorylated and total c-Myc levels. Cells were treated with 5 *μ*M SGI-1776, 5 *μ*M sunitinib, or the combination for 48 h. Phospho- and total c-Myc levels were detected by immunoblotting. Phospho- and total c-Myc levels were quantified by densitometry using ImageJ software. (**B**) Silencing c-Myc expression using siRNA. Cells were transfected with non-target control or c-Myc siRNA and c-Myc expression levels were determined by immunoblotting at 48 h. Band intensity was quantified using ImageJ software. (**C**) Knockdown of c-Myc enhances SGI-1776 and sunitinib-mediated apoptosis. 786-O cells were transfected with non-target control or c-Myc siRNA and then treated with 5 *μ*M SGI-1776, 5 *μ*M sunitinib, or the combination for 48 h. Apoptosis was determined by PI-FACS analysis. Mean±s.d., *n*=3. ^*^Indicates a significant difference compared with the non-target transfected cells treated under the same conditions. *P*<0.05.

To further evaluate the enhanced efficacy of PIM kinase inhibition in combination with sunitinib, we investigated the effects of this drug combination on c-Myc expression. Treatment with sunitinib alone did not significantly alter c-Myc phosphorylation. However, further reductions in c-Myc phosphorylation and total c-Myc levels were observed in combination-treated cells (Figure 4A). To determine whether drug-induced inhibition of c-Myc expression/activity is a critical determinant of the therapeutic efficacy of these agents, c-Myc levels were silenced using siRNA (Figure 4B) and treated with both single agents and the combination (Figure 4C). The anticancer activity of SGI-1776, sunitinib, and the combination were all significantly enhanced by c-Myc silencing, which suggests that modulation of c-Myc expression is a critical event underlying the efficacy of this combination.

### SGI-1776 augments the activity of sunitinib to reduce tumour burden in RCC xenografts

**Figure 5:**
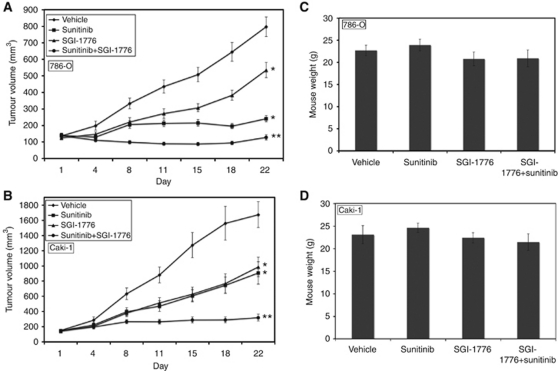
SGI-1776 enhances the activity of sunitinib in RCC xenograft models. (**A** and **B**) 786-O and Caki-1 cells (1 × 10^7^/mouse) were injected into the flanks of nude mice. When tumours reached ∼150 mm^3^ in size, mice were randomised into groups and treated on a QDx5 schedule with 200 mg kg^–1^ SGI-1776, 40 mg kg^–1^, or both agents for 3 weeks. Tumours were measured twice weekly. Mean±s.e.m., *n*=9. ^*^Indicates a significant difference compared with vehicle and ^**^represents a significant difference compared with either single-agent treatment. *P*<0.05. (**C** and **D**) SGI-1776 and sunitinib are well tolerated *in vivo*. Xenograft studies were carried out as described in Materials and methods. Animal body weight was determined at the end of the study (day 22) to quantify drug-induced weight loss. Mean±s.d., *n*=9.

To further investigate the potential benefit of the SGI-1776 and sunitinib combination, their activity was evaluated in two RCC xenograft models. 786-O and Caki-1 tumour-bearing animals were randomised into treatment groups and given 200 mg kg^–1^ SGI-1776, 40 mg kg^–1^ sunitinib, or the combination for 3 weeks on a QDx5 (every day for 5 days) schedule. Treatment with SGI-1776 or sunitinib alone resulted in a significant decrease in mean tumour volume in both xenograft models compared with the vehicle-treated controls (Figures 5A and B). Sunitinib elicited a more potent response in 786-O tumours compared with Caki-1, which may be due to the absence of VHL expression in 786-O. SGI-1776 dramatically enhanced the efficacy of sunitinib in both RCC models regardless of VHL status (Figures 5A and B). Importantly, all drug treatments were very well tolerated as no significant animal weight loss was observed at study completion (Figures 5C and D).

### SGI-1776 reduces Bad phosphorylation (Ser112) and induces apoptosis in RCC xenograft models

**Figure 6:**
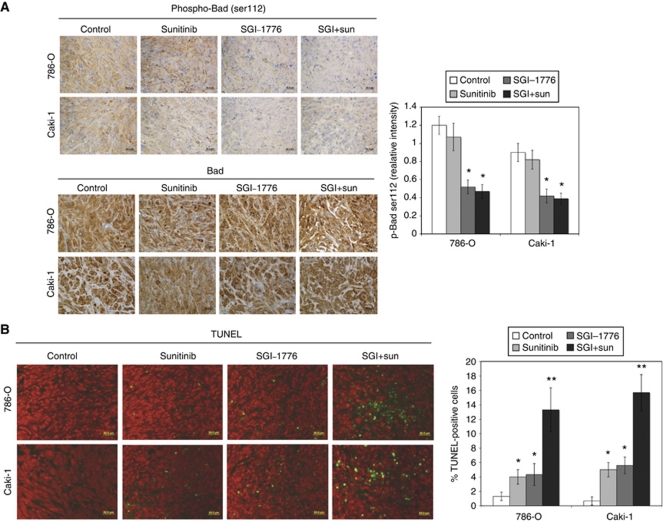
SGI-1776 reduces Bad phosphorylation and induces apoptosis in RCC tumours. (**A**) Phospho- and total Bad immunohistochemistry. Tumours were stained with either an antibody to phospho (Ser112) or total Bad and analysed by immunohistochemistry. The relative intensity of phospho-Bad expression was measured using Image-Pro Plus software Version 6.2.1. Mean±s.d., *n*=5. ^*^Indicates a significant difference compared with controls. *P*<0.05. No significant differences were observed in total Bad expression in the different treatment groups. (**B**) SGI-1776 and sunitinib induce apoptosis. Apoptosis was measured by TUNEL staining as described in Materials and methods. Quantification was conducted by manually counting TUNEL-positive cells. Mean±s.d., *n*=5. ^*^Indicates a significant difference compared with controls and ^**^represents a significant difference compared with single-agent treatments. *P*<0.05.

Since the phosphorylation of the pro-apoptotic protein Bad at Ser112 is a common marker of PIM kinase inhibition, its expression was evaluated in both tumours by immunohistochemistry. As expected, SGI-1776 induced a reduction in Bad phosphorylation without altering total Bad protein levels (Figure 6A). The addition of sunitinib did not alter the ability of SGI-1776 to decrease Bad phosphorylation. While SGI-1776 induced moderate levels of apoptosis, it significantly augmented sunitinib-mediated apoptosis in both xenograft models (Figure 6B). These data suggest that apoptosis induction adds to the anticancer efficacy of the SGI-1776 and sunitinib combination.

### Suppression of c-Myc levels is associated with a decrease in tumour cell proliferation

**Figure 7:**
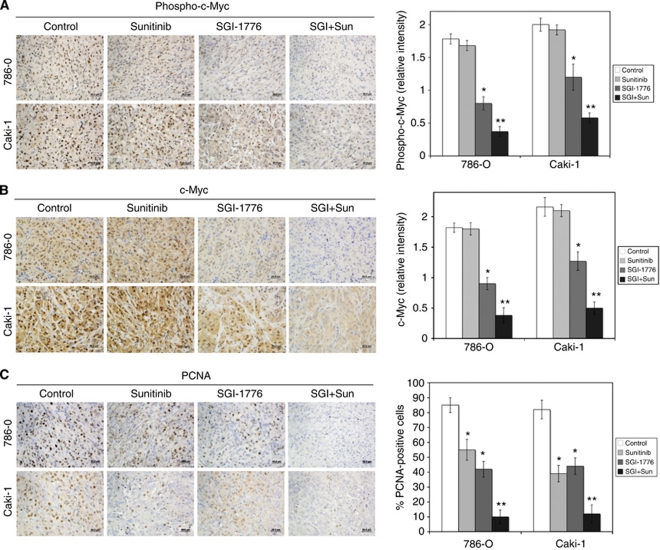
The combination of SGI-1776 and sunitinib significantly reduces c-Myc levels and decreases tumour cell proliferation. (**A** and **B**) The SGI-1776/sunitinib combination decreases c-Myc expression. Phospho- and total c-Myc levels were detected by immunohistochemistry. The relative intensities of phospho- and total c-Myc levels were measured using Image-Pro Plus software Version 6.2.1. Mean±s.d., *n*=5. ^*^Indicates a significant difference compared with controls and ^**^indicates a significant difference compared with single-agent treatments. *P*<0.05. (**C**) SGI-1776 and sunitinib reduce tumour cell proliferation. Tumour sections were stained with PCNA antibody. Positive cells were scored manually. Mean±s.d., *n*=5. ^*^Indicates a significant difference compared with controls and ^**^represents a significant difference compared with single-agent treatments. *P*<0.05.

Our *in vitro* studies demonstrated that modulation of c-Myc levels contributes to the activity of the SGI-1776/sunitinib combination. Immunohistochemistry was performed on the tumours to evaluate the effects of the drugs on phosphorylated and total c-Myc levels and tumour proliferation (PCNA). Consistent with our *in vitro* data, SGI-1776 decreased phospho- (Ser62) and total c-Myc levels in both tumour models (Figures 7A and B). The addition of sunitinib to SGI-1776 resulted in a further reduction in c-Myc levels, which was associated with decreased tumour cell proliferation (Figure 7C). Collectively, our data establish an important role for c-Myc in controlling RCC proliferation, which can be therapeutically targeted via PIM kinase inhibition to enhance the efficacy of the standard of care agent sunitinib.

## Discussion

Increased PIM kinase expression has been reported in many tumour types and is associated with tumourigenesis and drug resistance (Adam *et al*, 2006; Cibull *et al*, 2006; Beier *et al*, 2007; Popivanova *et al*, 2007; Hogan *et al*, 2008; Chen *et al*, 2009a). Consistent with this observation, PIM-1 levels were also elevated in RCC cell lines compared with RPTECs. Therefore, PIM-1 kinase activity may be a promising target for RCC. PIM-1 kinase phosphorylates the oncogene c-Myc, which strongly increases its stability (Zhang *et al*, 2008). c-Myc modulates the expression of a broad range of genes involved in cell-cycle progression, proliferation, metabolism, apoptosis, and angiogenesis (Pelengaris *et al*, 2002; Nilsson and Cleveland, 2003). While c-Myc overexpression occurs in a large percentage of tumours, it may have an especially important role in RCC due to the intrinsic hypoxic microenvironment that is characteristic of this tumour type (Tang *et al*, 2009). Furthermore, hypoxia has been reported to induce PIM-1 expression (Chen *et al*, 2009a). Consistent with c-Myc being a target for PIM-1, SGI-1776 treatment resulted in a reduction in c-Myc phosphorylation and total c-Myc protein levels. Thus, inhibition of PIM-1 kinase activity may be a novel approach to disrupt c-Myc function by promoting its degradation.

HIF*α* subunits are continuously being transcribed, but are targeted for proteasomal degradation under normoxic conditions by the VHL E3 ubiquitin ligase (Iliopoulos *et al*, 1996; Ohh *et al*, 2000). von Hippel-Lindau-defective tumours have high levels of HIF*α* and can be subdivided into two groups, tumours with both HIF-1*α* and HIF-2*α* (H1H2) and those with only HIF-2*α* (H2) (Gordan *et al*, 2008). HIF-1*α* and HIF-2*α* have opposing effects on c-Myc, where HIF-1*α* antagonises its activity and HIF-2*α* increases it. HIF-1*α* inhibits c-Myc activity by promoting its degradation via the proteasome and by binding to and activating Mxi-1, which represses c-Myc transcriptional activity (Zhang *et al*, 2007a). In contrast, H2 tumours are associated with high c-Myc activity and increased cellular proliferation compared with H1H2 tumours (Gordan *et al*, 2008). As expected, the H2 786-O cell line exhibited higher basal c-Myc levels than the VHL^+/+^ Caki-1 cell line in this study. However, c-Myc expression was detected in both cell lines, suggesting that PIM kinase inhibition may be an effective therapeutic strategy in both VHL^−/−^ and VHL^+/+^ tumours.

Recent studies demonstrate that PIM kinase expression results in chemoresistance to a number of agents with diverse mechanisms of action including cisplatin, taxane-based therapies, and rapamycin (Zemskova *et al*, 2008; Beharry *et al*, 2009; Hu *et al*, 2009; Mumenthaler *et al*, 2009; Chen *et al*, 2009a). In addition, it was recently demonstrated that treatment with docetaxel and cytarabine induced significant increases in PIM kinase expression (Zemskova *et al*, 2008; Kelly *et al*, 2011). Given this, PIM kinase inhibitors may have broad-spectrum activity across many malignancies and may be able to overcome resistance to multiple classes of anticancer agents with diverse mechanisms of action. Sunitinib continues to be utilised in first-line therapy for RCC and its activity has been mainly attributed to inhibition of tumour angiogenesis (Mendel *et al*, 2003; Abrams *et al*, 2003a, 2003b). However, several studies have also reported that sunitinib has antiproliferative and pro-apoptotic effects on tumour cells (Seandel *et al*, 2006; Xin *et al*, 2009). In our study, we demonstrated that sunitinib induces PIM-1 expression and that inhibition of PIM kinase activity with SGI-1776 significantly enhanced the efficacy of sunitinib in both *in vitro* and *in vivo* models of RCC. Interestingly, the SGI-1776/sunitinib combination led to a further reduction in c-Myc levels, which was associated with decreased tumour proliferation. A recent study showed that sunitinib downregulated c-Myc levels in acute myelogenous leukaemia cells, which was associated with monocytic differentiation (Nishioka *et al*, 2009). While we did not observe a strong effect of sunitinib on c-Myc levels in RCC cells, sunitinib was able to augment the ability of SGI-1776 to reduce c-Myc expression. In addition to c-Myc, PIM kinases have also been shown to phosphorylate the BH3-only protein Bad at Ser112, which inhibits its pro-apoptotic activity (Yan *et al*, 2003; Aho *et al*, 2004; Li *et al*, 2006). Recent studies in chronic lymphocytic leukaemia and prostate cancer are in agreement with our findings and demonstrate that SGI-1776 induces apoptosis (Mumenthaler *et al*, 2009; Chen *et al*, 2009b).

The combination of SGI-1776 and sunitinib dramatically reduced tumour burden in two RCC xenograft models compared with single-agent therapy and was very well tolerated. Sunitinib produced a more potent reduction of tumour burden in the 786-O cell line, which may be due to its VHL^−/−^ status. However, the SGI-1776/sunitinib combination was equally effective in both VHL^−/−^ and VHL^+/+^ xenograft models. Consistent with our *in vitro* data, analysis of the tumours revealed a decrease in c-Myc levels, which was associated with reduced tumour cell proliferation.

Our results suggest that targeting PIM kinase is a promising new strategy for the treatment of RCC. Its ability to reduce c-Myc activity may have important clinical implications since c-Myc overexpression is estimated to occur in 70% of human tumours. In addition, PIM kinase inhibition strongly enhances the activity of standard of care agents, such as sunitinib in the current study and platinum- and taxane-based therapies as reported by other investigators (Hu *et al*, 2009; Mumenthaler *et al*, 2009). Collectively, our data establish PIM kinase as a novel therapeutic target in RCC and provide the foundation for clinical investigation of PIM kinase inhibitors in combination with sunitinib.
